# Energy Efficient GNSS Signal Acquisition Using Singular Value Decomposition (SVD)

**DOI:** 10.3390/s18051586

**Published:** 2018-05-16

**Authors:** Juan Carlos Bermúdez Ordoñez, Rosa María Arnaldo Valdés, Fernando Gómez Comendador

**Affiliations:** School of Aeronautics and Space Engineering-Technical University of Madrid (UPM), Plaza Cardenal Cisneros 3, 28040 Madrid, Spain

**Keywords:** GNSS, GPS, compressed Sensing, sparse approximation

## Abstract

A significant challenge in global navigation satellite system (GNSS) signal processing is a requirement for a very high sampling rate. The recently-emerging compressed sensing (CS) theory makes processing GNSS signals at a low sampling rate possible if the signal has a sparse representation in a certain space. Based on CS and SVD theories, an algorithm for sampling GNSS signals at a rate much lower than the Nyquist rate and reconstructing the compressed signal is proposed in this research, which is validated after the output from that process still performs signal detection using the standard fast Fourier transform (FFT) parallel frequency space search acquisition. The sparse representation of the GNSS signal is the most important precondition for CS, by constructing a rectangular Toeplitz matrix (TZ) of the transmitted signal, calculating the left singular vectors using SVD from the TZ, to achieve sparse signal representation. Next, obtaining the M-dimensional observation vectors based on the left singular vectors of the SVD, which are equivalent to the sampler operator in standard compressive sensing theory, the signal can be sampled below the Nyquist rate, and can still be reconstructed via $\ell_{1}$ minimization with accuracy using convex optimization. As an added value, there is a GNSS signal acquisition enhancement effect by retaining the useful signal and filtering out noise by projecting the signal into the most significant proper orthogonal modes (PODs) which are the optimal distributions of signal power. The algorithm is validated with real recorded signals, and the results show that the proposed method is effective for sampling, reconstructing intermediate frequency (IF) GNSS signals in the time discrete domain.

## 1. Introduction

In software implementations, massive parallel correlation is done by exploiting the Fourier transformation. Mathematically, a time domain convolution is a multiplication in the frequency domain. By having all the IF samples in memory, we can transform to the frequency domain, perform a simple multiplication by the Fourier transform of the Pseudorandom noise (PRN) code, and later perform an inverse transform back to the time domain, this approach requires a large amount of random access memory RAM to store the data being received from the IF, and it is more of a store and process approach [1]. This research explores the use of compressed sensing (CS) to reduce the number of samples and, therefore, the amount of RAM required, which could allow the development of new processing signal technologies where the signal is processed where more computational resources are available.

Due to digital processing technology and the implementation of software-based GNSS receivers, researchers are motivated to try new acquisition and tracking methods of the GNSS signal with the advantages of robustness, sensitivity, and anti-jamming capabilities [2]. With the development of GNSS systems with more robust signals and the development of multiple constellations, GNSS receivers are facing a considerable amount of data processing, and the receiver hardware is growing larger, having a dramatic impact on the development of consumer- and professional-grade GNSS receivers. Receiver manufacturers are busily developing and implementing unique signal acquisition and tracking algorithms, advanced integrity monitoring algorithms, advanced multipath mitigation algorithms, and a host of other enhancements in an effort to improve the performance of GNSS receivers and make their products stand out in a crowded field [3]. The primary objective of this paper is to develop a technique that allows reducing the number of samples with a secondary goal of improve the computational requirements of GNSS signal acquisition by optimizing the computational complexity. For the purpose of this paper, acquisition its understood as the process to estimate the code phase and Doppler values of GNSS signals from the IF that are accurate enough to start tracking [4]. Thus, this paper focuses on a GNSS receiver in the cold start state when the receiver does not rely on stored information [5], specifically, Global Positioning System (GPS) receivers and its application to other constellations, like the European constellation Galileo.

**Figure 1:**
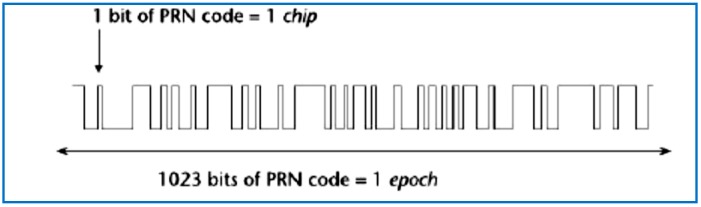
PRN code, chips, and epoch.

GPS receivers must observe and measure GNSS navigation signals from at least four satellites to obtain a three-dimensional position, velocity, and user clock error estimates. Use of more than the minimum four satellites will improve the accuracy of the user solution by using an overdetermined solution [6]. GPS satellites simultaneously transmit several ranging codes and navigation data using binary phase-shift keying (BPSK). However, only a limited number of central frequencies are used. Satellites using the same frequency are distinguished by using different ranging codes, also called chipping codes. Satellites are uniquely identified by a serial number called the space vehicle number (SVN) which does not change during its lifetime [7]. Additionally, all operating satellites have a pseudo-random noise (PRN) number which uniquely identifies the ranging codes that a satellite uses. The GPS satellite generates the signal. A frequency synthesizer driven by an atomic clock on the satellite makes a sinusoidal carrier frequency at 1575.42 MHz. This carrier is then modulated with a repeating code known as the C/A (coarse/acquisition) code. The C/A code is a binary sequence of 1023 bits, and it is used to multiply the carrier to form a binary phase-shift keyed (BPSK) modulated signal, The C/A is repeated every millisecond. The signal is further modulated by a 50-bps data stream containing the ephemeris data. It roughly takes 1 chip = 1 microsecond to travel the length of 300 m, and it takes 1 epoch = 1023 bits of PRN code 1 ms to travel 300 km (see Figure 1).

**Figure 2:**
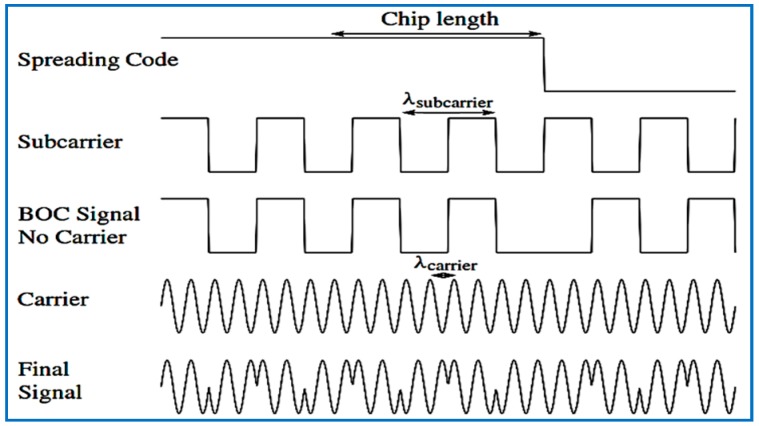
Spreading code, subcarrier, carrier, and signal as a result of the BOC modulation principle.

Galileo satellites transmit the E1 (L1) signal on the centered frequency 1575.42 MHz, the same as GPS and, with a reference bandwidth of 24.5520 MHz, the E1 signal contains pilot and data channels, and both use composite-binary offset carrier (CBOC) modulation(see Figure 2), which is multiplexed BOC(1,1) and BOC(6,1). 

The receiving power level at the Earth’s surface of r(t) is extremely weak, well below the noise floor. The minimum received power on the ground, defined at the output of an ideally-matched right-hand circularly-polarized 0 dBi user receiving antenna when the satellite elevation angle is higher than 10 degrees, is −157 dBW, considering 50%/50% E1B/E1C power-sharing [8].

Code A has 1023 MHz chipping rate, the data channel has a navigation message with 250 bps rate. The pilot channel is called E1-C, and the data channel is called E1-B. This kind of modulation allows GPS and Galileo signals to occupy the same frequency while avoiding mutual interference, making building receivers that use both GPS and Galileo simpler because GPS and Galileo use the same frequency.

A distinction is made between signals containing navigation data (the data channels) and signals carrying no data (pilot channels) [9]: the signals of the data and pilot channels are shifted by 90 degrees in phase, which allows for their separation in the receivers. Galileo allows the receiver to estimate the ionospheric delay error. This error is due to the delay that the navigation signals suffer when they travel through the ionosphere. This delay makes the distance from the satellite to the user, as measured by the receiver, appear longer than it actually is and, if not corrected, would lead to large positioning errors. Fortunately, this delay is proportional to the frequency of the signal, with lower frequency signals experiencing a longer delay than higher frequency signals. Therefore, by combining measurements to the same satellite at two different frequencies, it is possible to produce another measurement where the ionospheric delay error has been canceled out. This cancellation becomes more effective as the separation between the two frequencies increases. This is the reason why Galileo services are generally realized using pairs of signals [9].

**Figure 3:**
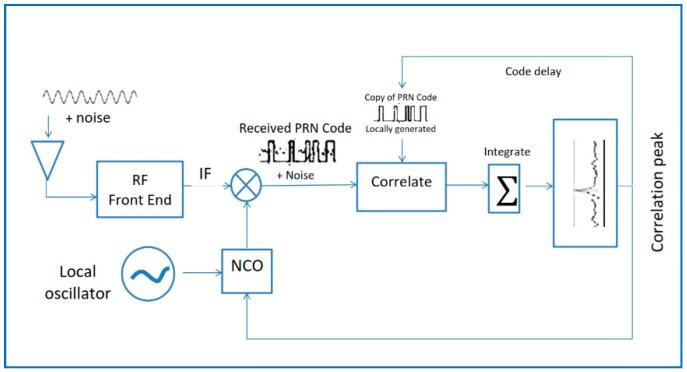
Basic GPS receiver architecture.

Basic GPS receiver architecture is shown in Figure 3. The satellite signal binary phase-shift keyed (BPKS) signal arrives at the antenna with some radio frequency (RF) plus noise. The front-end purpose of the receiver is to filter, amplify, and down-convert the incoming signal from analog to digital (A–D) to an intermediate frequency (IF) or lower frequency that is easy to process and sample in the receiver baseband. It is important to know that the RF front-end contains analog components that generate thermal noise and in the majority of satellite-receiver design the noise comes not from the satellites or any external source, but from the receiver itself [1]. After the front end, there is the baseband section of the receiver. The IF to baseband mixer acts to remove the carrier from the signal, leaving the original binary sequence that was created at the satellite and the 50-bps data, but also noise. 

At the correlator, the receiver takes a replica of the PRN code and multiplies it by the received signal, then integrates. When the correlators are aligned with the incoming signal a correlation peak is observed, and a hit is declared if the integrated value crosses a predetermined threshold. Moreover, the baseband block is repeated once per each channel so that each channel can acquire a different satellite. Therefore, a standard receiver has more than one channel. 

One aspect to notice is that until the correlation peak is found, there are two unknowns. One is the actual frequency offset by a Doppler value and the offset of the local oscillator at the receiver. Therefore, an important part is the acquisition space, which occurs in 2D, is that one axis is the frequency (KHz), and the other is the code delay (chips). The search is typically done in frequency bins. This is called a frequency and code-delay search. The traditional approach convolves then the received signal with the code division multiple access (CDMA) code of each satellite in the time domain, and the correct alignment corresponds to the one that maximizes convolution. This approach has a computational complexity of O$\left(n^{2}\right)$.

In the frequency domain the receiver takes the FFT of the received signal, it multiplies the output of this Fourier transform by the FFT of the CDMA code and then performs the inverse fast Fourier transform (IFFT) on the resulting signal, the output will spike at the correct shift that synchronizes the code with the received signal. The computational complexity of this approach is O(nlogn) [10].

Hassanieh et al. presented an FFT-based GPS locking algorithm of complexity O$\left(n\sqrt{logn}\right)$, called QuickSync, that builds on recent developments of sparse recovery, and introduces the lowest complexity algorithm to date. The algorithm is tested on two datasets with data collected in the US using an SDR and a second one collected in Europe. Their design reduces the number of multiplications for detecting the correct shift by a median of 2.2×, the algorithm aliases the received signal in the time domain before taking its FFT, performs a subsample FFT on the aliased signal, subsamples the FFT of the satellite CDMA code, and multiplies the resulting samples with the aliased subsample FFT. Then it performs the IFFT, and the output is aliased in the time domain. Picking the shift that maximizes the correlation [11], the algorithm developed in this research does not compete with the algorithms already in the market as its main focus is on compressing the signal that is to be used for those other algorithms.

Three contributors to the frequency offset to consider at the acquisition search are the frequency uncertainty and the noise in the TXCO-generated frequency, the Doppler effect for satellite motion, frequencies for rising and setting GPS satellites, and the receiver motion. For a receiver under static conditions, the most significant contributor to frequency offset is the satellite motion, which is about 4.2 KHz [1], however, under high dynamic conditions, signals produce significant Doppler frequency shifts, which hinders the fast acquisition of signals, in the case of the maximum velocity of the satellite combined with very high user velocity-approach values as high as 10 KHz [12].

The signal search and acquisition becomes important when the receiver is looking for several satellites at the same time: i.e., parallelism. A typical standalone GPS receiver can acquire signals down to about −160-decibel milliwatts (dBm) and might require a minute or more to obtain a position from a cold start. GPS receivers usually include some degree of parallelism, when considering a receiver having N channels, in which each channel is dedicated to searching for signals with a different PRN sequence. Within a channel, the frequency and code-phase search spaces are further divided into several windows [6].

Parallelism can be implemented in hardware using massively parallel correlators, or in software using fast Fourier transform-based techniques [13] where the massive parallel correlation is done by exploiting a property of the Fourier transformation. This approach requires having all the IF samples in RAM, where it can be transformed to the frequency domain, perform a simple multiplication, and finally perform an inverse transform back to the time domain. This will have the same results than using the standard hardware approach. However, due to the larger amount of data required to store the data received from the IF, this approach to store and process data requires a large amount of hardware or enough central processing unit (CPU) capacity.

Teixeira and Miralles developed a basic correlator using MATLAB and Simulink to validate the results and performance techniques when actual GPS satellite signal records are used, and formulated and implemented alternative parallel architectures to perform a circular correlation by decomposing the initial circular correlation into several smaller ones, which are independent and can be processed in parallel. When applied to GNSS signals, using FFT-based, parallel-code-phase search (PCS) has advantages for hardware-based implementations using field programmable gate arrays. The parallel architectures implemented are radix, FFTs, multipliers, adders, and NCOs. Additionally, the coded QuickSync algorithm, which exploits the sparse nature of the synchronization problem, and relays in an important property of aliasing a signal in the time domain, is equivalent to subsampling the signal spectrum [10]. The authors are in favor of software-defined radio (SDR) and the work presented provides a set of functional tools that allow to pretest initial prototypes of the GNSS-SVD-C algorithm.

The development of software-based GNSS receivers is rapidly revolutionized in satellite-based navigation applications, and the receiver technology needs to be updated efficiently for high positional accuracy requirements under noisy environments. As discussed before, the acquisition based on spread spectrum technology is an essential process for identifying satellites, with the development of GNSS and the emergence of multisystem joint positioning, the receiver design is moving towards more data processing and, therefore, hardware scale needs to be improved. The fundamental cause is that most of the sampled data is obtained by using the Nyquist-Shannon sampling theorem [14]. The theorem states that a signal can be exactly reproduced if it is sampled at a frequency F, where F is greater than twice the maximum frequency in the signal [15]. However, even though this is a sufficient condition for accurate recovery, it is not a necessary condition. This condition increases the system computation time and cost of modern wideband receivers. In a real application, sampling at the Nyquist rate usually produces a high number of samples. Additionally, the front-end design of future GNSS receivers must meet the needs of multi-navigation signal reception. Thus, the instantaneous bandwidth of RF front end is increased and increases the complexity of baseband signal processing [16]. The bandwidth of the receiver should be large enough to avoid signal to noise ratio (SNR) loss. This generally requires higher sampling rates with an attendant increase in power consumption and processing loads, a factor that is detrimental to low-cost and low-power consumer applications [6].

Song proposed a faster acquisition algorithm via subsample FFT. The algorithm first downsamples by a factor ‘*d*’ and then multiplies the FFT of the received signal with the FFT of the locally-generated PRN code, and takes the IFFT of the resulting signal, which produces a single spike at the correct time shift [17]. The problem with this algorithm is that the downsampling factor ‘*d*’ increases the noise contamination linearly, even though the computation time decreases exponentially, d log (d). The truncation of PRN sequences leads to a reduction in the correlation of the GPS signals and may not be an appropriate solution. Fortin and Landry identified GNSS signal characteristics and addressed them by a universal acquisition and tracking channel, proposing an architecture that allows sequential acquisition and tracking of any chipping rate, carrier frequency, FDMA channel, modulation—i.e., BPSK(q) or QPSK(q), sin/cos BOC(p, q), CBOC(r, p, Pr±), and TMBOC(r, p, $w_{r}$)—or constellation, where a mobile device could integrate fewer universal channels, securing signal availability and minimizing power consumption and chip size, the results showing a 66% increase in power consumption compared with the established reference [18]. The design principles align very well with this research in the sense that they identify the need to design new receivers to accommodate the increasing demands of new GNSS signals.

In recent years, the CS approach has been proven to effectively reduce the number of measurement samples required for digital signal acquisition systems. Compressed sensing, also known as compressive sensing, is a signal processing technique for efficiently acquiring and reconstructing a signal by finding solutions to underdetermined linear systems. This is based on the principle that, through optimization, the sparsity of a signal can be exploited to recover it from far fewer samples than required by the Shannon-Nyquist sampling theorem [19]. This research recommends an efficient method to acquire a GNSS signal using compressed sensing. Fortunately, the GPS signal, as any wireless RF signal, is relatively sparse [20]. The topic proposed in this paper is a novel CS method that requires low computation and regular hardware size, completes the acquisition process faster, and acquires weak signals until about −160 dBm.

An extensive description of CS theory is described in Section 2 and Section 3. The central problem of compressed sensing is the reconstruction of the high-dimensional sparse signal representation of x from a low-dimensional linear observation y.

A study from Hansen and Li performed a preliminary exploration of CS theory applied in GPS systems in 2012 [21]. They utilized the classic random binary matrix to observe the GPS signal and then adopt the reduced multiple measurement vector boost algorithm to reconstruct the signal. However, the signal reconstruction algorithm is very complex as the scheme is based on the multiple measurement CS theory. Kong proposed a two-stage compressed sensing algorithm taking a specifically structured matrix as the measurement matrix and employing multiple Walsh-Hadamard transforms as the signal reconstruction algorithm in 2012 [22], though the two-stage compressed sensing will lead to much higher algorithmic complexity. Additionally, the algorithm can be used only to acquire strong GPS signals, which is not always the case. 

Ou et al. developed a novel technique scheme based on CS achieves the transform sparsity of GNSS signals by utilizing the Gaussian random matrix and recovers the signal by using the single measurement OMP (orthogonal matching pursuit) algorithm [23]. This scheme has an extra carrier to noise(CNR) loss problem, and the extra CNR caused by the CS algorithm is inversely proportional to the compressed ratio. The research is useful in the sense that it implies how to select a better anti-noise performance measurement matrix and how to choose the best performance of a signal reconstruction algorithm based on different compression ratios, increasing the coherent integration and the number of non-coherent integration.

To solve the problem mentioned previously, a novel GNSS signal acquisition scheme based on compressed sensing is proposed in this research. The main focus is on $\ell_{1}$ minimization decoding models because $\ell_{1}$ minimization has the following two advantages: (a) the flexibility to incorporate prior information into decoding models; and (b) uniform recoverability [24]. A critical aspect regarding uniform recoverability is that recoverability is essentially invariant concerning different types of random matrices. This means that the random matrix does not have to be a random Gaussian or a random Bernoulli matrix with rather restrictive conditions, such as zero mean, and which are computationally expensive [22].

In real applications either measurements are noisy, signal sparsity is inexact, or both. Here inexact sparsity refers to the situation where a signal contains a small number of significant components in magnitude, while the magnitudes of the rest are small, but not necessarily zero. Such approximately sparse signals are compressible, too [24]. CS is an emerging methodology with a solid theoretical foundation that is still evolving. Most previous analyses in CS theory relied on the restrictive isometric property (RIP) of the measurement matrix A. These analyses can be called matrix-based. The non-RIP analysis, on the other hand, is subspace-based and utilizes the classic KGG (Kashin, Garnaev, and Gluskin) inequality to supply the order of recoverable sparsity [24].

Chang proposed a CS method to enhance GNSS signal acquisition performance with interference present. The interference is mitigated through the orthogonal feature between interference and the desired signal using the subspace projecting method. Meanwhile, the RIP can be preserved by projecting the Toeplitz-structured sensing matrix to ensure that the linear projection of the signal can retain its original structure and allow the recovery of the correlation output (sparse signal) [16]. This method is aligned with the topic in compressive sensing for this research, in the sense that it is subspace-based, but still uses the RIP approach to sound theory.

**Figure 4:**
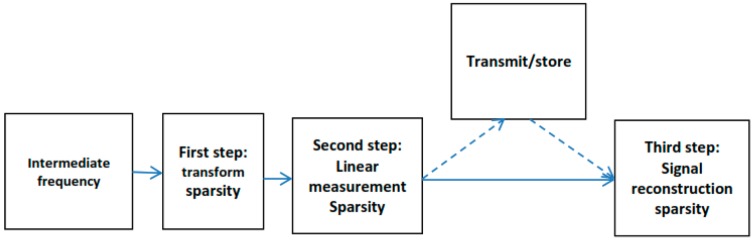
Signal processing based on compressed sensing.

The proposed CS model for the GNSS signal includes the three aspects shown in Figure 4. The first part is the sparse representation of the signal which consists of Toeplitz matrix design and sparse decomposition via matrix multiplication. The second part of this model is the compressed transmission; by linearly transforming the observation vector, the dimension can be reduced, which is far less than the original signal dimension. The third part is the reconstruction of the GNSS signal, since the observation vector can be calculated from the left singular and right singular vectors; the essence of the reconstruction is completed by using the convex relaxation method to match the original GNSS signal and, as part of this research, the GNSS-SVD-Convex algorithm is proposed to compress and reconstruct the signal.

## 2. Theoretical of Compressed Sensing

By focusing on the discrete-time where any instance of the input signal $x\;\in \;{\mathbb{R}}^{M}$ is represented by its Nyquist-rate samples. The CS framework is based on a sparsity assumption. The transform sparsity can be represented as the linear combination of a few vectors x=Ψs, where the transformation matrix Ψ is a given proper basis with the size of ${\mathbb{R}}^{M\times N}$, M represents the number of rows, and N represent number of columns. The coefficient vector s has only K≤M non-null coefficients [25]. However, due to white Gaussian noise $\upsilon \;\epsilon \;R^{N\times 1}$ present in real data, the transform sparsity step translates the signal x into the sparse signal. The observed linear measurements can be written as follows:

(1) y=Φx+υ=(ΦΨ)s+υ=As+υ

where $y\;\epsilon \;R^{N\times 1}$ is the measurement vector which represents each realization of x, A=ΦΨ is a N×N matrix that links the sparse representation s to y, and υ is additive noise modeling any random processes that occur in nature and non-idealities, and it is bounded by ${\left\|\upsilon \right\|}_{2}\leq \epsilon$, where $\Phi \;\epsilon \;{\mathbb{R}}^{N\times M}$ is a random Gaussian matrix. The compressed measurements are realized by a simple matrix multiplication and the ratio $R=\frac{N}{M}$ is generally called the compression ratio. Given that N<M, the reconstruction of the signal from y is an under-constrained problem. Then, the sparse coefficient s is recovered by solving the $\ell_{1}$ minimization problem for a given tolerance ϵ via convex optimization:

(2) $$\left(\ell_{r}^{1}\right):\;(\hat{s}\;\mathrm{to}min\;{\left\|s\right\|}_{\ell_{1}}\;\mathrm{subject to}\;\|\Phi \Psi s-{y\|}_{2}\leq \epsilon$$

where ${\left\|*\right\|}_{\ell_{1}}$ and ${\left\|*\right\|}_{\ell_{2}}$ represent the standard $\ell_{1}$ and $\ell_{2}$ norms, respectively, and ϵ considers the effect of noise υ. The right compressive ratio can be found via the energy packing property, as explained in Section 3 below.

### The Design of SVD-C-GNSS

It can take just milliseconds to measure the range to a satellite. However, it is the delay in the initial acquisition and the time required to decode ephemeris data that makes traditional GPS receivers slow to produce the first fix. For example, to make a position determination, a receiver must identify the code and then synchronize a local replica of it for at least three satellites. Four are necessary to remove receiver timing biases and track these signals for eighteen to thirty-seconds [26].

Given the lack of research directed towards the use of GNSS signal synchronization using fewer frequency samples, this paper expands the work described in Section 1 by attempting to investigate alternative methods of signal compression other than the traditional sparse Fourier transform. To this end the following goals are pursued:

1. Improve acquisition performance of the GNSS signal by using a compressive sensing algorithm based on $\ell_{1}$ minimization with non-restrictive isometric property RIP analysis. This method will allow finding the best anti-noise performance measurement matrix, given that it is not restricted to random Gaussian or random Bernoulli measurement matrices.
2. Develop a robust method for situations in which the receiver needs broader bandwidth to handle all types of navigation positioning signals using the non-RIP approach to compressive sensing which means the use of prior information to improve the acquisition stage.

The performance and advantages of these techniques will be shown based on recorded intermediate frequency (IF) real signals from a GPS front-end data loggers as an input to compressive sensing, where the incoming IF signal is sampled at an appropriate sampling frequency. The signal received by the antenna would go through amplification, mixing, filtering, an analog–digital conversion in the RF front-end, and its output is the IF signal [27]. 

The standard compressive theory contains three steps, including the transform sparsity of signal, the sparse signal with linear measurement, and the signal reconstruction [23].

High sample rates lead to high power consumption, which creates a hardware power consumption issue. A solution is presented here to lower the sample rates as much as possible and sample smarter by using techniques like low-rank matrix recovery for signal processing.

## 3. Singular Value Decomposition (SVD)

Here we introduce a useful concept, singular value decomposition, which is a method of decomposing a matrix, e.g., A into three other matrices. The SVD represents an expansion of the original data in a coordinate system where the covariance matrix is diagonal.

### 3.1. Theorem 1 

Let A be a n × d matrix with right singular vectors $v_{1}$,$\;v_{2}$, …,$\;v_{k}$, left singular vectors $u_{1}$,$\;u_{2}$, …,$\;u_{k}$, and corresponding singular values $\sigma_{1},\;\sigma_{2},\;\ldots ,\;\sigma_{k}$. Then A can be decomposed into a sum of rank one matrices as

(3) $$A=\overset{k}{\underset{i=1}{\sum }}\sigma_{i}u_{i}v_{i}^{T}\approx \sigma_{1}u_{1}v_{1}^{T}+\sigma_{2}u_{2}v_{2}^{T}+\ldots +\;\sigma_{k}u_{k}v_{k}^{T}=U\Sigma V^{T}$$

Proof: For each singular vector $v_{j}$, $Av_{j}=\sum_{i=1}^{k}\sigma_{i}u_{i}v_{i}^{T}v_{j}$. Since any vector v can be expressed as a linear combination of the singular vectors plus a vector perpendicular to the $v_{i}$, $Av=\sum_{i=1}^{k}\sigma_{i}u_{i}v_{i}^{T}v$, given two matrices are identical if for all vectors v Av=Bv, then $A=\sum_{i=1}^{k}\sigma_{i}u_{i}v_{i}^{T}$.

Suppose A is an m×n matrix whose entries come from field K, which is the field of real or complex numbers, then there exists a factorization called singular value decomposition of A, where the matrix *A* can be expressed as a sum of *k* outer product of vectors $\sigma_{1}u_{1}v_{1}^{T}+\sigma_{2}u_{2}v_{2}^{T}+\ldots +\;\sigma_{k}u_{k}v_{k}^{T},$ where $\sigma_{1}\geq \sigma_{2}\geq \sigma_{3}\ldots \geq \sigma_{n}\geq 0$. U is an m×n unitary matrix (if K=ℝ, unitary matrices are orthogonal matrices), V is an n×n unitary matrix over K, and $V^{T}$ is the conjugate transpose of V.

$\Sigma =U^{T}AV=\mathrm{diag}\left(\sigma_{1},\sigma_{2},\ldots ,\sigma_{p}\right)$ and the diagonal entries $\sigma_{i}$ of Σ are known as the singular values of A, where:

(4) $$\Sigma^{-1}=V^{T}A^{-1}U$$

U and V are orthogonal such that: $U^{T}U=I^{n\times n}$, and $V^{T}V=I^{n\times n}$, where I is the identity matrix.

**Figure 5:**
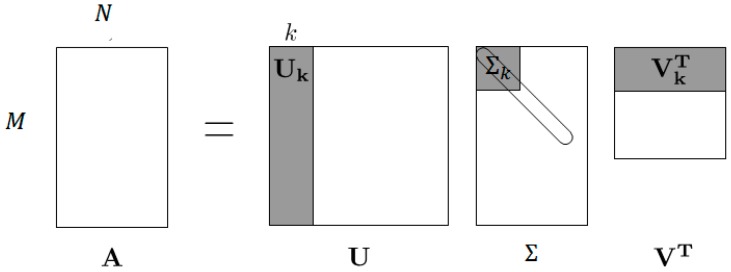
The singular value decomposition (SVD). Each singular value in *S* has an associated left singular vector in *U* and right singular vector in *V*.

Given the *m* × *n* matrix $A=U\Sigma V^{T}$ from Equation (3) and a target rank *k*≥1, it produces a rank-*k* approximation of A as follows. See also Figure 5.

1. Compute $A\;=\;U\Sigma V^{T}$.
2. Keep only the top k right singular vectors: set $V^{T}$ equal to the k rows of $V^{T}$ (a k × N matrix).
3. Keep only the top k left singular vectors: set $U_{k}$ equal to the first k columns of *U* (an M × k matrix).
4. Keep only the top *k* singular values: set $\Sigma_{k}$ equal to the first k rows and columns of Σ (a k × k matrix), corresponding to the k largest singular values of A.

The computed low-rank approximation is then:

(5) $$A_{k}=\;U_{k}{\;\Sigma \;}_{k}V_{k}{}^{T}$$

Storing the matrices on the right-hand side of takes O(k(n + d)) space, in contrast to the O(nd) space required to store the original matrix A. This is an enormous gain when k is relatively small, and n and d are relatively large [28].

In the matrix $A_{k}$, defined in Equation (5), all of the rows are linear combinations of the top k right singular vectors of A (with coefficients given by the rows of $U_{k}{\;\Sigma }_{k}$), and all of the columns are linear combinations of the top k left singular vectors of A (with coefficients given by the columns of $\Sigma_{k}$ $V_{k}{}^{T}$). Thus, $A_{k}$ has rank k, then, it might be possible to accurately recover a low-rank matrix from relatively few measurements.

### 3.2. SVD Properties

#### 3.2.1. Energy Packaging

SVD has the property of maximum energy packing. This property is usually used in compression [29], and it is a stable method to split the system into a set of linearly independent components, each of them bearing their own energy contribution. SVD offers a low-rank approximation which could be an optimal sub-rank approximation by considering the largest singular value that packs most of the energy contained in the signal and representing the matrix A as truncated matrix k, which allows storing matrix A as an approximation of matrix $A_{k}$.

#### 3.2.2. Noise Filtering

By zeroing out small singular values (large $\Sigma_{i}^{-1}$) we can low pass filter the input vector *x*, which gives the opportunity to filter out noise in the measurement.

### 3.3. Sensing

SVD is constituted from two orthogonal dominant and subdominant subspaces. The only part of *x* that matters is the component that lies in the N-dimensional subspace of $R^{N}$ spanned by the first columns of V. Thus, the addition of any components that lies in the null-space of A will make no difference.

In standard compression sensing, to recover the signal, a priori knowledge of the seed that generates Φ is required, and the dictionary Ψ [30], which could be a circular matrix or particular kind of Toeplitz matrix is, in this paper, the seed, and the dictionary comes as a first approximation of the signal by using a non-symmetric Toeplitz matrix that replaces the conventional random sensing matrix. We will call it the Toeplitz matrix or dictionary.

The matrix $Tz\in {\mathbb{C}}^{m\times n}$ is called the Toeplitz matrix if each diagonal parallel to the principal diagonal is constant. In mathematical terms:

(6) $$\forall \;a_{i,j\;}\in T\to a_{i,j\;}=a_{i+1,j+1}$$

(7) $$Tz=\left[\begin{array}{c} \begin{array}{cc} x_{0} & \begin{array}{cc} x_{1} & x_{n} \end{array}\\ x_{1} & \begin{array}{cc} x_{0} & x_{1} \end{array}\\ x_{2} & \begin{array}{cc} x_{1} & x_{0} \end{array} \end{array}\\ \begin{array}{c} \begin{array}{ccc} x_{3} & x_{2} & x_{1} \end{array}\\ \begin{array}{ccc} x_{m} & x_{3} & x_{2} \end{array} \end{array} \end{array}\right]$$

Once the Toeplitz dictionary is established, and the SVD computed, the signal can be compressed by using the *U* left singular vector (LSCs) of the GNSS signal, which are the eigenvectors of $XX^{T}$. The compression is done by multiplying the transpose of U to the observed signal, $U^{T}x$, where T stands for transpose of the matrix.

### 3.4. GNSS SVD Compressed Sensing Scheme

The method proposed for the acquisition of GNSS signals is to use SVD for sensing the signal and $\ell_{1}$ minimization for matrix recovery which can be expressed in the following sum of terms form, where $a_{0},a_{1},a_{2},\ldots ,a_{m-1}$ are the coefficients of the basis vectors, and, where the signal can be defined on an orthonormal basis:

(8) $$f\left(x\right)=a_{0}+a_{1}x+a_{2}x_{2}+a_{3}x_{3}+\ldots +a_{m-1}x_{m-1}$$

Let $x\;\epsilon \;R^{M}$ be a GNSS signal and let $\Psi =\left\{\Psi_{1},\;\Psi_{2},\ldots ,\Psi_{M}\right\}$ be a basis vector spanning $R^{M\times N}$. The discrete time signal can be represented sparsely as:

(9) $$\hat{x}=\overset{M}{\underset{i=1}{\sum }}\theta_{i}\Psi_{i}=\Psi \theta$$

where $\theta \;\epsilon \;R^{d}$ is a coefficient vector of $\hat{x}$ in the Ψ domain. If θ is sparse, then the solution to an undetermined system of the form x=Ψθ, $\theta \;{\mathbb{R}}^{N\times 1}$ where the unknowns d are greater than the observations *M* that can be solved using $\ell_{o}$ minimization, but this problem is NP-hard [31].

Defining the sensing matrix A=ΦΨ, $A\in R^{M\times N}$ let p=min(M,N), K≤r be the number of non-zero singular values of *A*:

(10) Ax=y

(11) $$U\Sigma \underbrace{V^{T}x}=y$$

Multiplying both sides by $U^{T}$, where the superscript “*T*” means the “transpose” of matrix U, $U^{T}U=I$:

(12) $$I\Sigma \underbrace{V^{T}x}=\underbrace{U^{T}y}$$

where $\hat{x}=\underbrace{V^{T}x}$, and $\hat{y}=U^{T}y$:

(13) $$\Sigma \hat{x}\;=\;\hat{y}$$

where A is the sum of *k* rank-one matrices, $\hat{y}$ is the measurement vector and, for some scalars, $\sigma_{1},\sigma_{2}$, $\sigma_{3},\;$…, $\sigma_{k}$ ≥ 0 and orthonormal vectors $u_{1},\;u_{1},\ldots ,u_{k}\in R^{M}$ and $v_{1}$, $v_{2}$, **…,** $v_{k}$ ∈ $\;R^{N}$. The {$\sigma_{k}$} can be interpreted as the k largest singular values of *A*, and the $\left\{u_{i}\right\},\;\left\{v_{i}\right\}$ as the corresponding singular vectors. The collection of all such matrices form a union of subspaces in $R^{M\times N\;}$; each set of vectors $\left\{u_{i}\right\},\;\left\{v_{i}\right\}$ define an R-dimensional subspace, and the {$\sigma_{k}$} correspond to an expansion in that subspace. Since x can be represented sparsely as θ in the Toeplitz dictionary, and x is known, the desired sparse solution can be recovered by using Equation (14):

(14) $$\left(\ell_{k}^{1}\right):\;\hat{\theta }\;\mathrm{to}\;min{\left\|\theta \right\|}_{\ell_{1}}\;\mathrm{subject to}\left(\theta_{i}-\frac{y_{i}}{\sigma_{i}}\right)\leq \epsilon ,\;i=1,2,\ldots ,p$$

However, due to noise (white Gaussian) v $\in \;R^{N}$ present in real data, x may not be expressed as a sparse superposition of *s*, and Equation (9) needs to be modified to:

(15) $$\hat{x}=\Psi \theta +v$$

where ║*v*║_2_ is bounded by ${\left\|v\right\|}_{2}$. The sparse θ can still be recovered accurately by solving the stable $\ell_{1}$ minimization problem via the second-order cone programming [32] using Equation (2).

### 3.5. Proper Orthogonal Modes (POD)

One useful measurement is the proper orthogonal modes (POD) which are the optimal distributions of signal power; the calculation of the POD by using modal projection is done with the following loop:

(16) $$\mathrm{For}\;j=1:k\;A_{k}=u\left(:,1:j\right)\times \sigma \left(1:j,1:j\right)\times v{\left(:,1:j\right)}^{T}\;\mathrm{End}$$

The loop above processes the sum of the first to k modes, resulting in the modes of interest, the energy in each mode and the POD approximation is computed in the following manner:

(17) sig=diagonal(σ)

(18) $$energy\left(r\right)=\frac{sig\left(1:k\right)}{sum\left(sig\right)}$$

**Figure 6:**
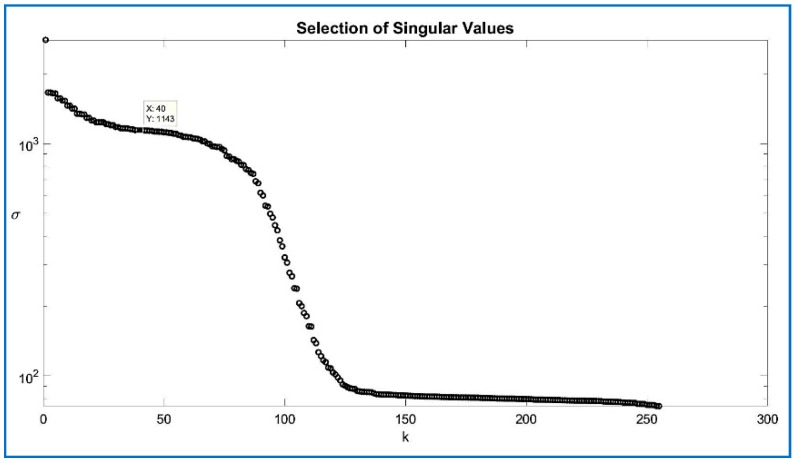
Scree plot.

Alternatively, by computing the log of the diagonal singular values $\sigma_{ii}$. Regarding the selection of PODs, a well-known solution based on a scree plot was developed by Cattell [33] as shown in Figure 6. The PODs can be graphically found by localizing the inflection point on the semi-log scale where the PODs remain flat, and where the sloppy line and the flatline intersect (“elbow”). After plotting the entire spectrum of singular values, it is expected a clear dominance of the first modes, those are the columns of the matrix U or column space, and constitute the orthonormal expansion basis of interest, where $\sigma_{ii}$ are the first k singular values of interest.

### 3.6. Algorithm

In terms of algorithms to solve convex problems, one approach has been used in this paper and is explicitly specified where needed; as a general guideline, once a convex formulation of a problem is found, testing it with the aid of modeling languages, such as CVX [34], allow its solution by means of general solvers that handle linear or quadratic programming [35].

Recent advances in algorithms for solving convex optimization problems, along with significant advances in processor power, have dramatically reduced solution times. Perhaps more exciting is the possibility that convex optimization can be embedded directly in signal processing algorithms that run online, with strict real-time deadlines, even at rates of tens of kilohertz [34]. The automatic code generator discovers the sparsity, and calculates how to exploit it, at code-generation time.

CVX is a MATLAB-based modeling system for convex optimization. CVX turns MATLAB into a modeling language, allowing constraints and objectives to be specified using standard MATLAB expression syntax. For this paper the use of CVX (a package for specifying and solving convex programs [36,37]) in the MATLAB programing language was used to solve Equation (19) and to run Algorithm 1.

(19) $$min{\left\|\theta \right\|}_{\ell_{1}}S.T.\;\|\theta_{i}-{\frac{y_{i}}{\sigma_{i}}\|}_{\ell_{2}}\leq \epsilon ,\;i=1,2,\ldots ,p$$

**Algorithm 1.** Compressive sensing GNSS-SVD-C.*Input:*Measurements are segmented into m×n vector of length m
$x_{b1}=\left[x_{0},x_{1},\ldots ,x_{M}\right]$*Steps*
Compute the desired compression ratio *R* = $\frac{N}{M}$ R ∈(0,1) to establish input N columns for the Toeplitz matrix after establishing the soft threshold from the scree plotConstruct the Rectangular Toeplitz Matrix $Tz=\left\{\left[x_{0},x_{1},\ldots ,x_{M}\right],{\left[x_{0},x_{1},\ldots ,x_{N}\right]}^{T}\right\}$Compute the SVD for the Toeplitz matrix output arguments:U−Left Singular Vectors(Matrix)V−Right Singular Vectors(Matrix)Σ−Singular Values(Diagonal Matrix)Compress the signal $y=U^{T}x_{b1}$ for all subsequent x in buckets of the same size M e.g., *x*$b2=\left[x_{M+1},x_{M+2},\ldots ,x_{K=M}\right]$Calculate the sparse vector signal using $\ell_{1}-\mathrm{minimization}$ by solving the convex optimization problem of Equation (19).*Return:* Once θ is computed, the original signal x is decoded by computing $x=\Psi V\theta =U\Sigma V^{T}V\theta$ for each one of the buckets or windows compressed on step 7. By using the proposed method, only a small set of measurements is required to recover the vector $x\;\in \;{\mathbb{R}}^{M\times 1}$.

## 4. Simulation and Performance

This study is conducted using three sets of raw GPS data, which are processed after running the algorithm on SoftGNSS [5], a state of art software-defined Global Positioning System (GPS) receiver whose performance is improved by a dual-frequency approach, which, for this paper, is considered as the ground-truth. When considering the presented results it is important to notice the difference between outputs from the GNSS SVD-C compressed sensing algorithm and the state of the art software, the aim is to have similar performance, but with significantly fewer observations. The algorithm’s purpose is to attain the best solution regarding data size, which is a key parameter on standalone battery-operated applications.

### 4.1. Performance Metrics

To evaluate the performance of the proposed compression scheme, several objective tests were made. Factors such as the signal to noise ratio (SNR), computational complexity, probability of detection, probability of false alarm and graphical comparison of the execution time of each operation were computed.

#### 4.1.1. Signal to Noise Ratio (SNR)

The signal to noise ratio is defined as the ratio of signal power to the noise power. A higher SNR means the signal quality is better. It is measured in decibels (dB). The signal to noise ratio is defined by:

(20) $$SNR=10\;log_{10}\left[\frac{\sum_{n=0}^{N-1}x{\left(n\right)}^{2}}{\sum_{n=0}^{N-1}{\left[x\left(n\right)-y\left(n\right)\right\}}^{2}}\right]$$

where x(n) is the original signal, y(n) is the compressed and recovered signal, and n is the length of the signal. The low SNR levels, especially below 20 dB, have a significant impact on the sparse approximation process, and the higher measurement noise contributes to either low peak sharpness or inaccurate recovery [38]. For this research, a SNR above 20 dB after signal recovery is considered successful.

#### 4.1.2. Computational Complexity

The initial step of forming the Toeplitz matrix requires a complexity of $O(N^{2}$) operations, and the economy SVD rank reduction step requires $O(N^{2}k$) operations. The multiplication of the left singular vectors with the transmitted signal is of size (r−N) × *N*. The convex relaxation requires a complexity of $O(N^{3}$) operations. In the worst case the complexity of the whole algorithm is $O(N^{3}$), and this is still within an acceptable range.

#### 4.1.3. Acquisition Time

One of the performance metrics on this research is to use a generalization of Holmes’ method [39], where the time from when the receiver is turned on to when the user solution is obtained, or the first position fix (TTFF) metric, is subdivided into different contributions, distinguishing among three scenarios: cold, warm, and hot start. TTFF depends on the status of the receiver, the availability and validity of the data required to compute the navigation solution, the carrier to noise ratio $C/N_{0}$, the number of visible satellites, the receiver method of processing on all the signals from the visible satellites [40], and the influence of the ionosphere, tropospheric refraction, multipath and many other sources of error. This metric is a crucial factor in GNSS receiver design because it is perceived as the primary performance characteristic in the mass-market for receivers:

$$TTFF_{cold}=T_{warm\;up}+T_{acq}+T_{track}+T_{CED}+T_{GST}+T_{PVT}$$

where $T_{warm\;up}$ is the receiver warm-up time; $T_{acq}$ is the acquisition time; $T_{track}$ is the settling time for code and carrier tracking; $T_{CED}$ is the navigation data read time (clock correction and ephemeris data); $T_{GST}$ is the GNSS system time; and $T_{PVT}$ is the time to compute the navigation solution.

By focusing on reducing the acquisition time $T_{acq}$, the total TTFF can be decreased by using new algorithms and technology. The proposed algorithm will have an impact on $T_{acq}$ by reducing the number of samples. 

#### 4.1.4. Probability of Detection (Pd) and Probability of False Alarm (Pfa)

The analysis of the probabilities of detection and false alarm is closed to the analysis done for the L1 C/A signal in [1].

The result of the circular correlation between the receiver and the local signal in Figure 3 can be modeled as:

(21) $$I_{i}\left(\tau_{u},f_{du}\right)=Ad_{i}R\left(\tau -\tau_{u}\right)sinc\left(\left(f_{d}-f_{du}\right)T_{1}\right)\cos\left(\theta_{ei}\right)+nI_{i}$$

(22) $$Q_{i}\left(\tau_{u},f_{du}\right)=Ad_{i}R\left(\tau -\tau_{u}\right)sinc\left(\left(f_{d}-f_{du}\right)T_{1}\right)\sin\left(\theta_{ei}\right)+nQ_{i}$$

where $\tau_{u}$ and $f_{d}$ are the possible code delay and Doppler shift, in the same manner τ and $f_{d}$ are the true code delay and Doppler shift, and d is a data bit value. R(.) is the autocorrelation function of the C/A code, T is the millisecond correlation interval, $\theta_{e}$ is the average phase error over the integration time, A is the amplitude, which is normalized to derive the noise variance to 1. The terms $nI_{i}$ and $nQ_{i}$ are the in phase and quadrature components of the noise, and both have the distribution $N\left(0,\;\frac{\sigma_{N}}{\sqrt{2}}\right)$, where $\sigma_{N}^{2}$ is the total noise power at the input to the correlation.

To determine the correct alignment, a threshold is chosen above the noise power that has a low probability of being exceeded by the noise. That probability is called the probability of false alarm (pf), and the computation of pf is straightforward by constructing a Gaussian distribution centered at the mean value of the noise and computing the area under the tail of the distribution.

Considering the results after non-coherent integration, a function of the form

(23) $$r=\sqrt{I^{2}+Q^{2}}.$$

If a signal is present r has a Rayleigh distribution with mean and variance given by [41]:

(24) $$\mu \left(X\right)=\sigma \sqrt{\frac{\pi }{2}}$$

(25) $$var\left(X\right)=\frac{4-\pi }{2}\sigma^{2}$$

The approach to compute the probability of detection is also to construct a Gaussian distribution centered around the n peak, and computing the area under the curve that is above the false alarm threshold once the threshold is established for the pf. The standard deviation at the peak is different than the standard deviation away from the peak by the definition of SNR [1] as the power ratio of the peak magnitude to the noise standard deviation, $\sigma_{N}$. For a given SNR the variance at the peak comes from the Rice distribution and is given by the following equation from [1,41]:

(26) $$\sigma_{P}^{2}=\nu^{2}+2\sigma_{N0}^{2}-{\langle V\rangle }^{2}$$

where $\nu =S_{0}$, the mean amplitude of the coherent peak is the standard deviation of the noise on *I* or *Q* $\sigma_{N0}$; 〈V〉 = mean $\left(S+\mu_{N}\right)$:

(27) 〈V〉=(σN0π2)e−γ2[(1+γ)I0(γ2)+γI1(γ2)]

where $I_{0}$ and $I_{1}$ are the *n*th-order modified Bessel function.

The variance away from the peak as Equation (26) is, σN2=σN02(4−π)2, the ratio of the variances at the peak and away from the peak is:

(28) σP2σN2=ν2+2σN02−〈V〉2σN02(4−π)2

(29) q=σP2σN2=44−π(γ+1−π4e−γ[(1+γ)I0(γ2)+γI1(γ2)]2)

(30) γ=S02σN02

where γ is the coherent SNR, and q is the ratio of the standard deviation at the correlation peak to the standard deviation away from the peak. The correct alignment is then determined by the delay in an output that exceeds the threshold when a signal is present and it is called the probability of detection.

In practice, to compute the probability of detection (Pd) this research assumes that because the non-coherent integration comprises the sum of many samples, the resulting probability distribution is close to Gaussian, derived from the central limit theorem [1] and the bell-shaped curve centered at the expected value of the correlation peak after non-coherent integration, and the probability of detection can then be computed by calculating the area under the curve and above the threshold and using Equation (29).

## 5. Numerical Results

Several simulation experiments have been conducted using MATLAB R2016b (The MathWorks, Inc., Natick, MA, USA), under Windows 7, on a regular PC 64-bit operating system, with an Intel^®^ Core™i5-4200U CPU @ 2.30 GHz, to verify the feasibility of the GNSS signal compression scheme described above. The simulations are executed in three parts: the GPS C/A signal compression with the static receiver, the BOC^1^ signal compression with the static receiver, and the GPS C/A signal compression for receiving under avionic conditions. All experiments used real data recordings, and cover both urban and suburban areas.

### 5.1. Datasets

The datasets were created using two signal records from the book “A Software Defined GPS and Galileo Receiver”, the files: GPSdata-DiscreteComponents-fs38_192-if9_55.bin (collected at the University of Colorado, Boulder, CO, USA), GPS_and_GIOVE_A-NN-fs16_3676-if4_1304.bin (collected in Turin, Italy), and a third dataset Feb6.u8.bin (collected in Randsburg, CA, USA). Interestingly enough, the algorithm was validated with real GNSS-recorded data under avionic conditions for the receiver. Dataset 3 contains GNSS data from a high-power rocket flight that captured GPS RF data for post-processing. A description of the data is presented now in Table 1, and the parameters necessary for processing the datasets are as follows:

The C/A code repeats every millisecond, but the data packets are modulated by the C/A code every 50 bps, and there is a possibility of a bit transition every 20 ms; therefore, a 1 ms chunk of data is reliable for satellite acquisition and is widely adopted in practice [5].

To ensure good probability of successful acquisition 763,840 samples from Dataset 1 were divided into 10 segments of 76,384 samples, and each segment was segmented into 40 vectors, each of a length of 1910. Thus, we have *m* = 1910, *n* = 40 and *t* = 76,400. 

For illustration purposes, after an acquisition using the algorithm with 2.5% ($\frac{1910}{76,400}$) or compressed measurements R = 0.025 which means the Toeplitz matrix has N = 40 columns for Dataset 1, results showed the signal is acquired by the acquisition software, and results match the ones in the book “A Software-Defined GPS and Galileo Receiver”. Regarding the validation of the algorithm, PRN 21 is present, since the SoftGNSS Version 2 software detects this satellite, it validates the developed algorithm. Additionally, the book indicates that the file already mentioned does not include PRN 19, the results from SoftGNSS does not detect it either, but detects all the PRN as stated in the book. Results from Table 2, Table 3 and Table 4 below show the output from SoftGNSS for IF without the application of the compression GNSS-SVD-C algorithm.

For Dataset 3 the received GPS ‘L1’ signal from the radio frequency (RF) front end is converted to an intermediate frequency (IF) of 4.1304 MHz and sampled at a frequency of 16.367 MHz for 1 ms of data, the number of samples can be found as 1/1000 of the sampling frequency, i.e., 16.367 × 10^6^ × 1/1000 = 16,367 samples. To ensure good probability of successful acquisition, we have confined the value to 32,734 samples.

**Figure 7:**
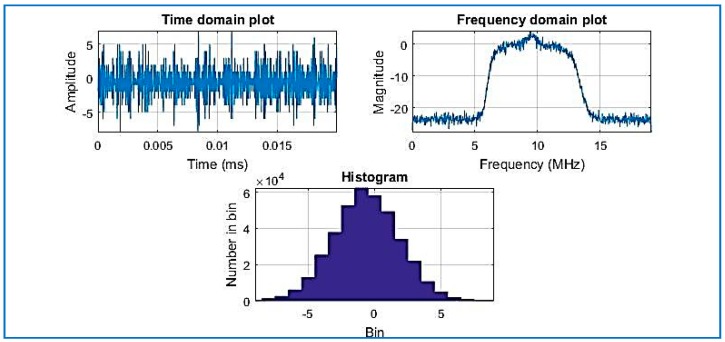
Compressed signal with R = 0.025, Dataset 1.

**Figure 8:**
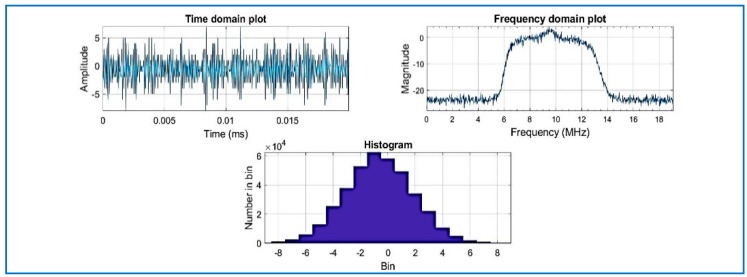
Raw IF data plotted signal of Dataset 1 (not compressed).

Figure 7 shows estimated output of power spectral density (PSD) plots. The PSD algorithm is using the fast Fourier transform (FFT)) for the acquisition [5]. Observe the histogram in Figure 7 where the amplitude of the compressed signal fluctuates mostly between the values of 6 and −6: the result is the same as Figure 8, where the same dataset is used (without compression) and processed by the same software (SoftGNSS). The results show that after the signal is compressed by the algorithm, the signal is detected successfully after the changes imposed by the algorithm.

**Figure 9:**
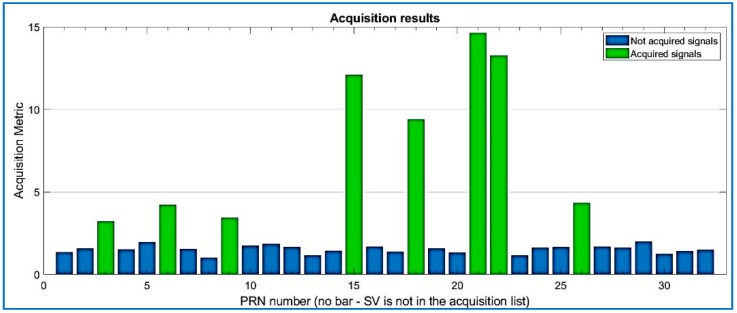
Acquired PRNs from Dataset 1, with R = 0.025.

**Figure 10:**
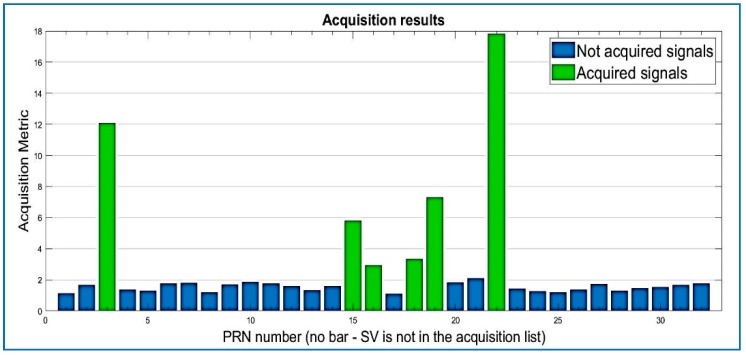
Acquired PRNs from Dataset 2. R = 0.03.

**Figure 11:**
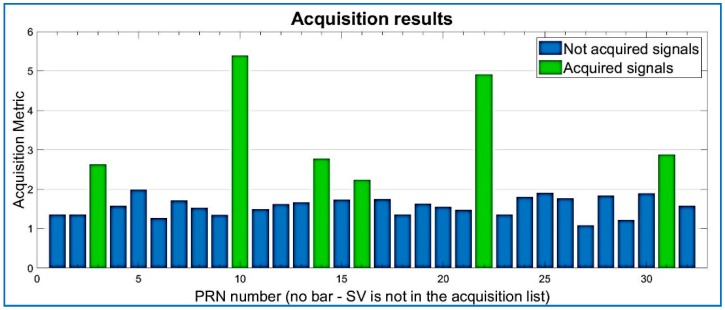
Acquired PRNs from the RTLSDR receiver/Dataset 3. R = 0.30.

Results for the compressed signal are depicted below in Figure 9, and the quality of the acquisition is the same performance as the non-compressed signal, for the same dataset. Regarding Datasets 2 and 3, after data compression and recovery, the satellites are also acquired by the state of the art software, as shown in Figure 9, Figure 10 and Figure 11.

**Figure 12:**
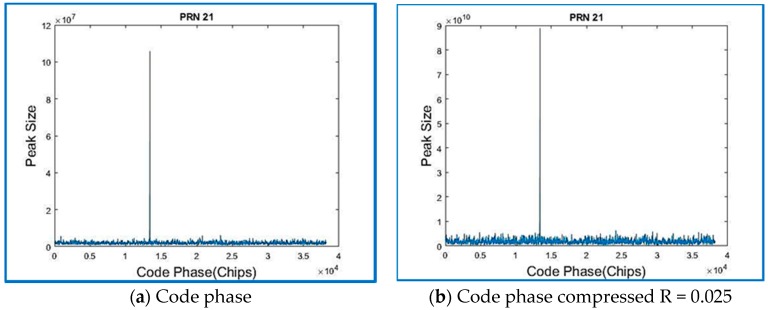
Recorded real signal from Dataset 1: (**a**) the code phase; and (**b**) the code phase of the GPS signal when the signal is compressed.

Figure 12 shows the correlation outputs for a signal that was acquired using a regular method, which is a parallel code phase search, with a Doppler search step: 500 Hz and 2 ms data length (corresponding to 1 PRN) sampled at Fs = 38.192 MHz. The acquisition is successful when a satellite is visible, and one is provided with a coarse estimation of the carrier frequency of the GPS raw signal, as well as its code phase. In theory, only one dominant peak should be observed at the correct code phase–frequency bin combination. Peaks of smaller magnitude may coexist due to signal and noise interference [38].

**Figure 13:**
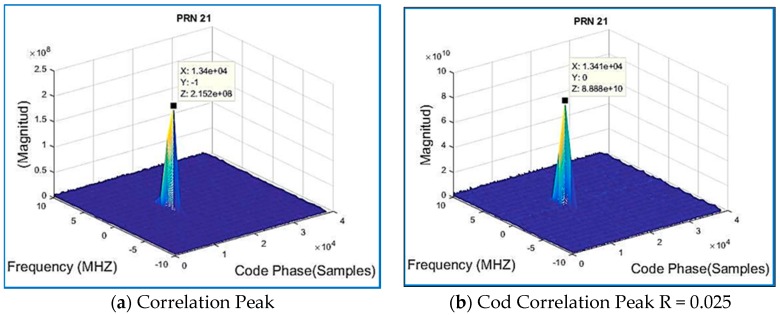
Recorded real signal from Dataset 1: (**a**) the correlation peak; and (**b**) the correlation peak of the regular GPS signal compressed for the same Dataset 1.

**Figure 14:**
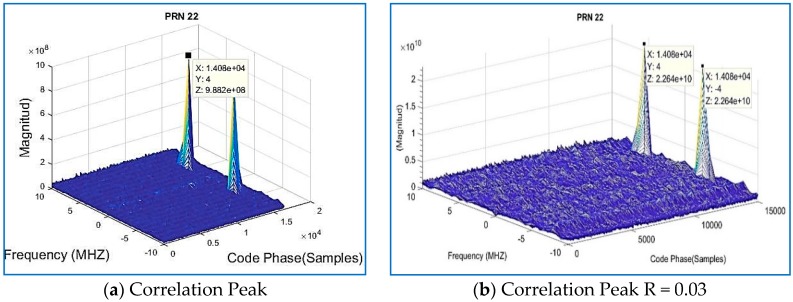
Recorded real signal Dataset 2: (**a**) the correlation peak; and (**b**) the correlation peak of the GPS signal when the signal is compressed with R = 0.03.

Traditional methods for acquisition performance assume the satellite is acquired if a certain threshold is obtained. The computation of the metric is obtained by SoftGNSS by dividing the maximum peak coefficient by the second highest correlation peak in the same frequency bin, and that threshold was set to 2.5. The correlation peak is shown in Figure 13a,b, and by comparing both figures the peak size can be observed as larger by two orders of magnitude for the compressed sensing signal. Figure 13 reverberates what it was described before in Section 3.2, the most dominant coefficients are the only useful information, and it is representative of the signal’s time delay and Doppler shift. Observe how, for the compressed signal, the correlation peak is of much greater magnitude, 8.88 × 10^10^ vs. 2.15 × 10^8^ (for raw data from Dataset 1), and the frequency is centered on the zero-frequency bin. Similar results were obtained for Dataset 2 on Figure 14.

**Figure 15:**
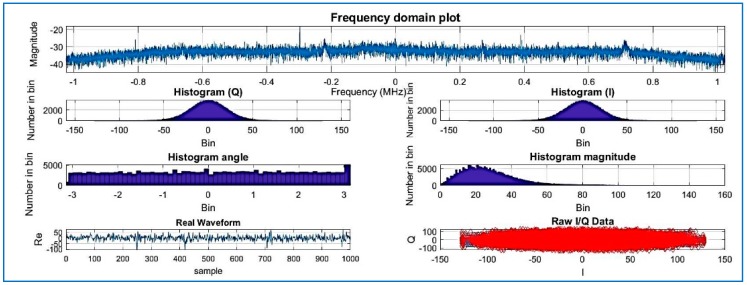
Frequency domain, histogram, real waveform, and raw I/Q of Dataset 3.

**Figure 16:**
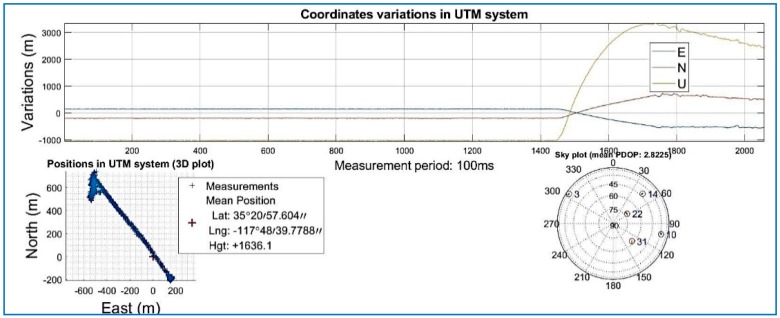
Navigation solution of Dataset 3 (not compressed).

**Figure 17:**
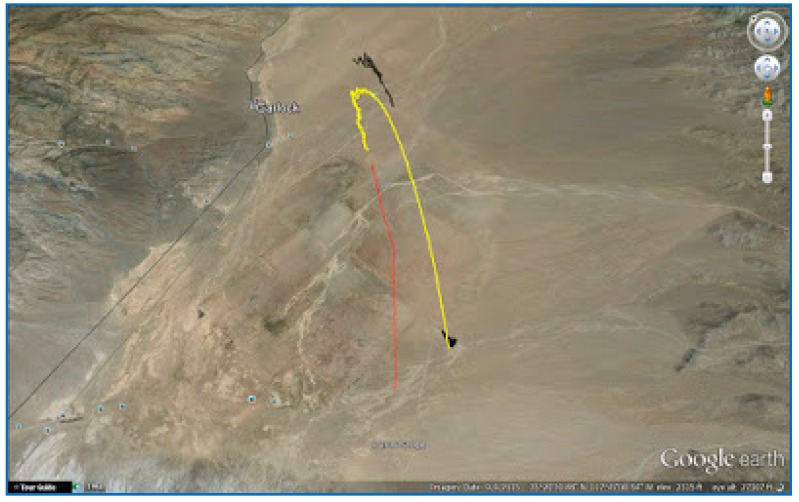
KLM file for Google Earth, Dataset 3. Source: [43].

The SoftGNSS code is flexible enough to work with a variety of file formats, including MATLAB “uchar” 8 [44] unsigned integers. For Dataset 3 the signal is processed before compressing in SoftGNSS and the results can be seen in Figure 15, Figure 16 and Figure 17 where the output from the post-processing module of SoftGNSS is shown, as well as the Keyhole Markup Language (KLM) file for Google Earth.

**Figure 18:**
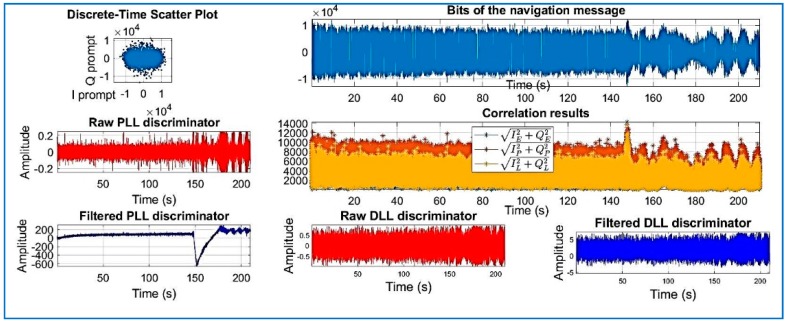
PRN 10, channel 01, Dataset 3.

**Figure 19:**
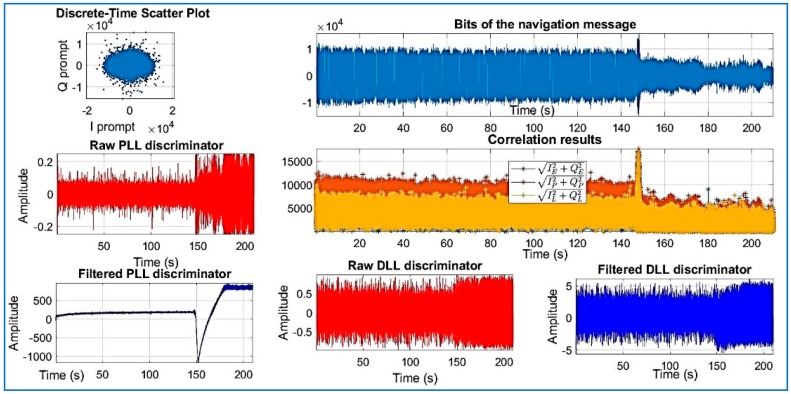
PRN 22, channel 02, Dataset 3.

**Figure 20:**
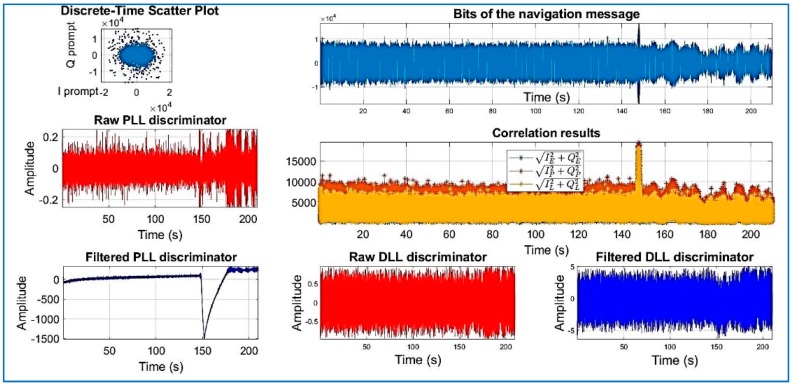
PRN 31, channel 03, Dataset 3.

**Figure 21:**
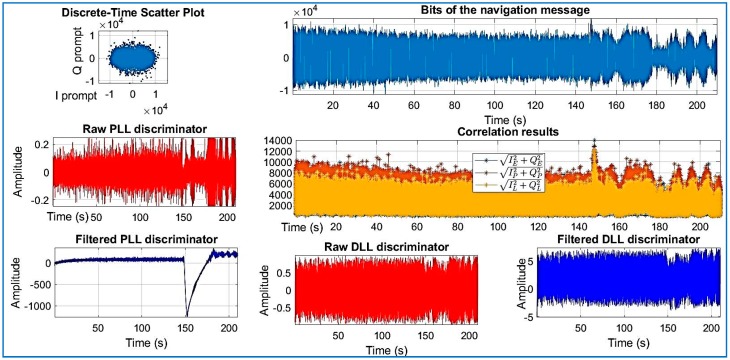
PRN 14, channel 04, Dataset 3.

**Figure 22:**
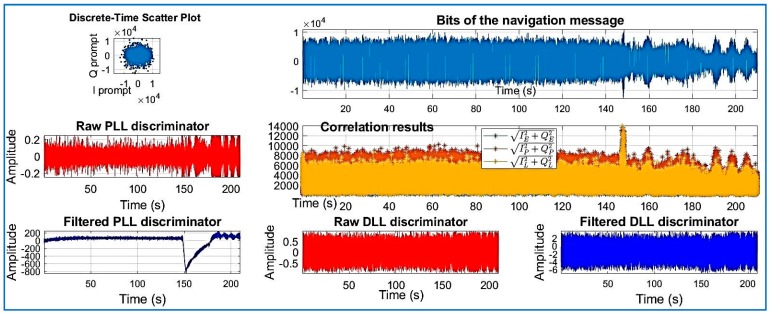
PRN 03, channel 05, Dataset 3.

**Figure 23:**
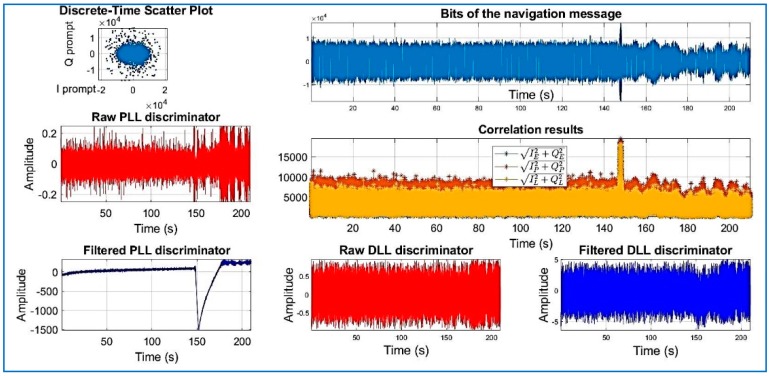
PRN 31, channel 03, Dataset 3.

**Figure 24:**
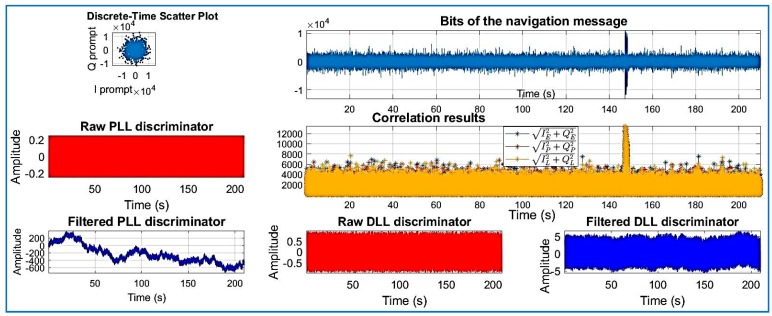
PRN 16, channel 6, Dataset 3. Observe how the raw discriminator is saturate. The software discarded the PRN after processing.

Figure 18, Figure 19, Figure 20, Figure 21, Figure 22, Figure 23 and Figure 24 show the output from the SoftGNSS software for raw GNSS Dataset 3.

### 5.2. Compression Performance

The diagonal values are said to make up the singular value spectrum, and the importance of the singular values are given by its magnitude. To be more specific, the square root of each singular [45] value is proportional to the variance explained by each singular vector [46]. Assuming that small PODs are related to noise, we can use this assumption to reduce noise [46].

Table 5 below compares the quality of the GNSS-SVD-C algorithm among several compression levels, as there is a direct relation with the PODs. Results show that increasing the compression increases the SNR, improving the quality of the signal, and the optimal value has to be calculated. The best approach is to determine the N number of columns from the scree plot (see Figure 6) and stop at that soft threshold. As can be seen, N = 40 columns for the Toeplitz matrix gives a SNR = 29.31 with an acquisition time of 2.70 s. The number of columns represent the rank of the TZ matrix and the number of $y=U^{T}x$ compressed items for a given bucket on the compression algorithm. By increasing the number of columns, the acquisition time increases with no significant noise reduction, but with an increase in computational time. The increase of the peak size is relevant if the noise floor remains the same. From Table 5, the noise floor or bed can be inferred, increasing with increasing peak values which explains why the higher peaks reached by CS do not correspond with an increase in the SNR, in that sense the use of PODs are more relevant to obtain an optimal SNR. Another method to know if the algorithm enhances the acquisition is to compare both methods against the probability of detection.

**Figure 25:**
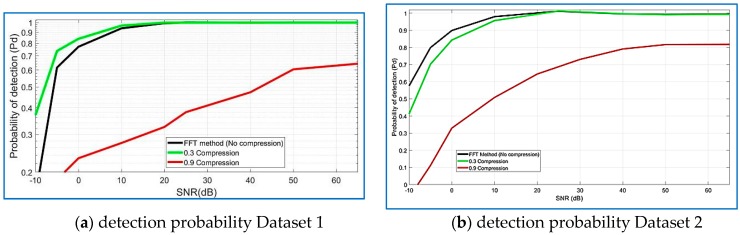
Distribution of the detection probability for several SNRs, Dataset 1 and Dataset 2.

According to Figure 25a, the detection probability of the GNSS-SVD-C algorithm is similar to that of the FFT parallel code search algorithm with high SNR, which are both close to 1, and the false alarm probability is $10^{-3}$. The detection probability of the FFT parallel code search algorithm reduces rapidly along the decreasing SNR, while that of GNSS-SVD-C algorithm with R = 0.30 just reduces slowly. In addition, the detection probability of the FFT parallel code search algorithm deteriorates with the low SNR, and that of the GNSS-SVD-C also reduces rapidly, but lower than GNSS-SVD-C. Both algorithms perform the same above 20 dB with R = 0.30 for GNSS-SVD-C. FFT parallel code search algorithm performs better than GNSS-SVD-C when R = 0.90.

**Figure 26:**
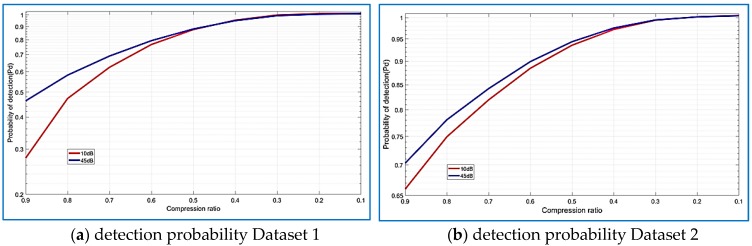
Distribution of the detection probability and compression ratio for several SNRs, Dataset 2.

Similar results are achieved in Figure 25b for the GPS and Giove satellites, the detection probability of the GNSS-SVD-C algorithm with R = 0.30 is 100% on the 20 dB SNR, close to the FFT parallel code search algorithm. FFT parallel code search algorithm performs better than GNSS-SVD-C when R = 0.90. According to Figure 26a,b the detection probability of the GNSS-SVD-C algorithm increases when the SNR increases and produces the best results for compression ratios from 0.10 to 0.30. 

## 6. Conclusions and Future Scope

A novel GNSS signal acquisition algorithm based on CS and SVD is proposed aiming to reduce the computational complexity of GPS and BOC satellite signals. A methodology is presented to choose the effective compression ratio by using a scree plot in combination with the probability of detection.

The algorithm enhances the input for the baseband and provides a simple dimensionality reduction mechanism to condense the dataset. The SVD-based sensing of GNSS signals approach is dependent on finding the economy SVD of the autocorrelation trajectory matrix of noisy input samples (Toeplitz) and maintaining the structure of the matrix by applying suitable convex relaxation methods. The main idea was to use a Toeplitz matrix with the time-shifted reference signal as the dictionary that leads to a sparser representation.

When testing with recorded real GNSS signals, this method achieves the same results of the conventional regular detection method for SNR’s above 20 dB, with implicit signal filtering and within an acceptable mean acquisition time. The detection of the number of visible satellites is maintained, and the re-acquisition of GPS data is avoided. At the same time, the use of SVD to sample the GNSS signals where random matrices (Gaussian) may not be the best choice, the combined GNNS-SVD-C algorithm offers a good approach to signal energy-oriented low-rank approximation to GNSS signal reconstruction. The theoretical foundation of this work is based on non-traditional compressive sensing as we do not adhere to the strict RIP condition. As explained in Section 1, RIP is only a sufficient, but not a necessary condition, for reconstruction accuracy; therefore, a stable solution is still recoverable by $\ell_{1}\;\mathrm{minimization}$.

The methodology also allows to sense the signal at the front end and store it in the time domain and/or transmit it for processing where more computational resources are available. For delay-tolerant applications, offloading GPS signals for processing to the cloud or to base stations is possible. GNNS-SVD-C is a CS approach that will limit the associated costs in transfer operations, and the sparse representation based GPS acquisition technique can efficiently capture and embed information in a lower-dimensional space and, subsequently, recover it from an underdetermined system where the criteria to design the measurement basis may take advantage of a priori knowledge of the signals to acquire.

Our work in this paper is guided by the current hardware limitations of low-cost and low-power sensor platforms. We believe that the key observations and principles derived here will find their way to applications in acquisition systems that have constrained hardware resources to handle the bulk of data processing. Further, we believe that the algorithm we introduce has other applications in signal processing. We plan to explore those applications in future work. 

## Figures and Tables

**Table 1:** Datasets.

Data Set	File Name/Reference	Sample Frequency (MHz)	Intermediate Frequency	Signed Character	Doppler Frequency Search
1	GPSdata-DiscreteComponents-fs38_192-if9_55.bin/ [42]	38.192	9.55 MHz	Bit8	±10 kHz
2	GPS_and_GIOVE_A-NN-fs16_3676-if4_1304.bin/ [42]	16.3676	4.1304 MHz	Bit8	±10 kHz
3	Feb6.u8.bin/ [43]	2.048	2210.53 Hz	uchar	±10 kHz

**Table 2:** PRNs of Dataset 1.

Channel	PRN	Frequency	Doppler	Code Offset
1	21	9.54742 × 10^6^	−583	13,404
2	22	9.54992 × 10^6^	1921	6288
3	15	9.54992 × 10^6^	1921	36,321
4	18	9.54843 × 10^6^	428	20,724
5	26	9.54492 × 10^6^	−3078	26,827
6	6	9.54443 × 10^6^	−3569	28,202
7	9	9.55092 × 10^6^	2923	4696
8	3	9.54992 × 10^6^	1921	34,212

**Table 3:** PRNs of Dataset 2.

Channel	PRN	Frequency	Doppler	Code Offset
1	22	4.13468 × 10^6^	4277	14,077
2	03	4.13440 × 10^6^	4004	7363
3	19	4.13694 × 10^6^	6541	6341
4	15	4.13209 × 10^6^	1686	1492
5	18	4.13247 × 10^6^	2069	1528
6	16	4.13125 × 10^6^	851	2071

**Table 4:** PRNs of Dataset 3.

Channel	PRN	Frequency	Doppler	Code Offset
1	10	2.39844 × 10^3^	188	1523
2	22	3.90625 × 10	−2171	1680
3	31	−1.03906 × 10^3^	−3250	512
4	14	2.31250 × 10^3^	102	358
5	03	−2.76563 × 10^3^	−4976	1729
6 *	16	2.30398 × 10^5^	228,188	1252

* Could not find valid preambles in channel 6.

**Table 5:** Mean detection time and SNR, Dataset 1.

Columns	Peak Size	Noise Floor Power	Mean Detection Time	SNR
5	3.49 × 10^8^	3.30 × 10^14^	2.65	22.60
10	1.00 × 10^9^	3.29 × 10^15^	2.70	25.48
20	4.55 × 10^9^	5.93 × 10^16^	2.73	23.43
30	1.11 × 10^10^	3.88 × 10^17^	2.70	24.39
40	2.08 × 10^10^	1.37 × 10^18^	2.70	29.31
50	3.27 × 10^10^	3.10 × 10^18^	2.79	22.95
80	8.73 × 10^10^	1.77 × 10^19^	2.76	22.37
150	3.09 × 10^11^	2.73 × 10^20^	4.31	27.82
300	1.15 × 10^12^	4.42 × 10^21^	14.97	28.10
350	1.62 × 10^12^	9.04 × 10^21^	144.09	23.89
